# A mixed methods study to evaluate the feasibility of using the Adolescent Diabetes Needs Assessment Tool App in paediatric diabetes care in preparation for a longitudinal cohort study

**DOI:** 10.1186/s40814-017-0164-5

**Published:** 2017-07-06

**Authors:** Helen Cooper, Gillian A. Lancaster, Phillip Gichuru, Matthew Peak

**Affiliations:** 1 Department of Public Health and Wellbeing, Clatterbridge Hospital, University of Chester, Clatterbridge Rd, Bebington, Birkenhead, Wirral CH63 4JYL UK; 2 Research Institute for Primary Care & Health Sciences, Keele University, Keele, ST5 5BG UK; 3 Department of Mathematics and Statistics, University of Lancaster, Lancaster, LA1 4YF UK; 4 NIHR Alder Hey Clinical Research Facility, Alder Hey Children’s NHS Foundation Trust, Eaton Road, West Derby, Liverpool, L12 2AP UK; 5 Department of Public Health and Wellbeing, University of Chester, Riverside Campus, Castle Drive, Chester, CH1 1SL UK

**Keywords:** Evaluation, Type 1 diabetes, Needs assessment, Questionnaire, Patient education, Glycaemic control, App

## Abstract

**Background:**

An evaluation study was carried out to determine the feasibility of integrating the Adolescent Diabetes Needs Assessment Tool (ADNAT) App into UK paediatric diabetes care, to ascertain best practice standards and to determine methodological recommendations for a future cohort study.

**Methods:**

A non-randomised, cohort, mixed methods study design was used to ensure equality of access to ADNAT for all participants at three sites in the North West of England. Following UK Medical Research Council guidance, the RE-AIM (reach, effectiveness (potential and perceived), adoption, implementation, maintenance) framework was used to guide study objectives and feasibility outcomes. Patients who completed ADNAT (completers) were compared with those who failed to complete (non-completers). Patients’ glycaemic control (HbA_1c_) was accessed from their clinical data at baseline and at 6 months, alongside their ADNAT scores which were correlated with changes in HbA1c levels. The diabetes teams (respondents) completed a web-based survey and attended focus group interviews.

**Results:**

Eighty-nine patients were recruited. Withdrawal rates were low at 4.5% (*n* = 4). Forty-four patients (49.4%) completed ADNAT, leaving 45 (50.6%) non-completers. There were large baseline differences in HbA1c and variable rates of change at 6 months. After adjusting for baseline HbA_1C_ and site in an analysis of covariance, completers had a lower post-ADNAT mean HbA_1C_ level than non-completers at 6 months (-5.42 mmol/mol, 95% CI −11.48, 0.64). Patients’ glycaemic control (HbA_1c_) at 6 months correlated reasonably well with their ADNAT scores (Spearman’s rho = 0.46). Survey and focus group data showed that ADNAT was judged to be an effective clinical tool by the diabetes teams. Value to patients was perceived by the teams to be linked to parental support, age and previous diabetes education. The combined data triangulated. It served to capture different dimensions which were used to define changes to achieve practice standards and methodological recommendations.

**Conclusions:**

The combined data showed that ADNAT has the potential to be a clinically viable tool. It has demonstrated the need for a randomised design that is tailored for a ‘hard to reach’ adolescent population. A cluster randomised controlled trial that involves sequential but random rollout of ADNAT over multiple time periods may be the most appropriate and is currently being considered for the larger study.

**Trial registration:**

NIHR Children’s Clinical Research Network, UKCRN ID 6633

**Electronic supplementary material:**

The online version of this article (doi:10.1186/s40814-017-0164-5) contains supplementary material, which is available to authorized users.

## Introduction

This paper reports on a study which evaluated the feasibility of using the Adolescent Diabetes Needs Assessment Tool (ADNAT) App in three Paediatric Diabetes Units (PDUs) in the North West of England. The study used a realist evaluation approach [1] to address two issues: firstly, whether the ADNAT App could be integrated into paediatric diabetes care and, secondly, to determine best practice standards and methodological recommendations for a future cohort study. A core assumption of a realist perspective is that phenomena such as ADNAT are complex interventions introduced into constantly changing systems, which has particular relevance to paediatric diabetes care in the UK. Comparisons within and between clinical sites were therefore required to determine what did and did not work and why, in order to establish local modifications necessary to ensure effectiveness in practice. This relationship is expressed in Medical Research Council (MRC) guidance [2] for process evaluation of complex interventions which defines evaluation of context, implementation and mechanisms of implementation as the primary aims of such studies.

## Background

Type 1 diabetes (T1D) is one of the most common endocrine and metabolic conditions in childhood within Europe. The UK, which has 27,600 children and young people living with the condition, alongside the Russian Federation and Germany, makes the largest contribution to the overall numbers in T1D in young people with an incidence rate of 25.9 per 100,000 of the general population aged 0–15 years and a prevalence rate of 195.4 per 100,000 [3, 4]. Alongside this, young people in the UK also have one of the worst rates of glycaemic control in Europe, which is associated with later micro- and macrovascular risk [5]. This has been demonstrated in successive National Paediatric Diabetes Audits (NPDA) for England and Wales for those aged 0–25 years, with the latest for 2015–2016 [4] reporting improving but still disturbing figures alongside the need to reduce variability in outcomes:

- Only 26.6% achieved recommended glycaemic targets of less than 58 mmol/mol, with 17.9% having levels above 80 putting them at high risk of complications.
- Glycaemic variability is due to service related factors, including standards and delivery of diabetes self-care education which showed wide Paediatric Diabetes Unit (PDU) level variability with 30% receiving no structured education.
- For those aged 12 years and over: 26.3% had high blood pressure, 19.7% had high total cholesterol, 9.7% had albuminuria (sign of kidney disease), over 13.8% had early signs of eye disease, and 18.1% were overweight and 20.8% were obese.
- Overall, just 35.5% of children and young people received all seven of the recommended key care processes including blood glucose (HbA1c), body mass index, blood pressure, urinary albumin, cholesterol, eye screening and foot examination.
- In general, those in the most deprived areas, were of non-white ethnicity, were adolescent, or female had poorer outcomes.

These findings have been supported by the UK’s National Peer Review Programme [6] which highlighted inequity of service provision to young people. They also fit with the UK’s Kennedy Review [7] which described teenagers as a ‘forgotten group’, reporting that their health care needs are given low priority by commissioners, policymakers and clinicians alike, and recommended investment in youth friendly services. This is particularly important since many adolescents lack the skills and strategies to avoid behaviours that carry health risks which has specific relevance to those with diabetes given their added risk of future debilitating complications.

In 2012, the UK government responded to the problem by introducing a paediatric diabetes ‘Best Practice Tariff’ (BPT) which defines 13 mandatory care standards including tailored education [8]. For adults, over a 10-year period, such education has been shown to save the National Health Service (NHS) £2200 per patient, breaking even at 4 years [9, 10]. No such data are available for young people, although Swift [11] reported that education for young people has greater effects than for adults with small to medium effects on glycaemic control and larger effects on psychosocial outcomes.

Given these changes, instigated by the BPT, alongside the annual National Paediatric Diabetes Audit [4], and a Peer Review Quality Assurance Programme [6], paediatric diabetes clinical practice was undergoing extensive changes. These emerging changes meant that ‘routine care’ was neither standardised nor constant at the time of the study so that the outcomes of using ADNAT depended upon clinical context and health professionals’ responses to its implementation, making the setting a mediator of outcomes.

In the UK, six paediatric educational trial interventions have been completed [12–16]. They all followed traditional didactic face-to-face approaches, reported considerable variations in outcomes, and no significant long-term changes in glycaemic control. Recommendations included the need to review research methodology and to modernise paediatric care through the use of technology enhanced learning (TEL) to support long-term patient training. This latter recommendation is supported by a review of technology enabled approaches to diabetes management which endorsed self-assessment tools and tailored education based on patients’ unique histories and their immediate needs [17]. In support of this, a meta-analysis of 46 studies found that a blend of TEL and face-to-face instruction had stronger learning outcomes than did face-to-face instruction alone in primary/secondary/tertiary education [18]. However, there are few validated diabetes websites for young people, the majority being directed toward adults; there is wide variation in the quality of evidence provided, and they offer didactic information at high reading levels with little problem-solving assistance [19, 20]. Social networking, as a patient-led tool, is growing in popularity and starting to be used by patients and practitioners but research in children with T1D in all these areas is lacking, both in terms of quantity and quality, reflecting the complex issues of using social media as a clinical tool [21]. Systematic reviews [22, 23], including our own [24], have consistently highlighted an absence of rigorous UK-based research, minimal use of theory and no reporting of process, health inequalities, dose response and cost-effectiveness data. In addition, findings highlighted the need to personalise learning in alignment with developmental stages, i.e. age-related reasoning and cognitive abilities, making regular needs assessment a core requirement. No instrument to assess such needs was located in the UK. We therefore developed, validated and psychometrically tested the ADNAT App. The App provides secure username and password protected access to ADNAT through mobile devices, e.g. smart phones and tablets, delivers immediate feedback to users and emails confidential patient data to practitioners.

### The ADNAT App

Development, validation and psychometric testing of ADNAT have been reported elsewhere [25–27]. The research programme^,^ followed Medical Research Council (MRC) guidance for complex interventions [28]. It has included studies of adolescent diabetes self-care [29–31] and technological methods of learning [24] and theory [32]. ADNAT combines reflective questioning with needs assessment to raise self-awareness to support adolescent decision-making in relation to diabetes self-care. It consists of 117 questions divided between six domains including all about me, physical activity, eating, monitoring blood glucose, medication taking and living with diabetes. The number of questions answered by users is filtered according to, for example, insulin regimen and lifestyle factors. Thirty-six of the questions, hidden amongst the total, provide self-care and psychosocial health scores Needs Assessment Ratings (NARs) (the self-care questions with examples of some of the shorter range of responses are shown later in Table 6). Classical test theory and item response analysis validated the use of simple additive scores which we translated into traffic light responses. Scoring for each item was 0 for green, 1 for amber and 2 for red responses, so that high scores indicated high need, moderate scores intermediate need and low scores low need.

From our previous research which qualitatively analysed the perceptions of the clinical usefulness of ADNAT with young people, parents and health professionals [26], ADNAT was theoretically determined to have the following mechanisms of action:

1. Mediator for facilitating tailored education and support by raising patients’ self-awareness about their diabetes self-care and coping mechanisms, identifying patient-led foci for conversation in the clinical consultation and providing practitioners and patients with data to guide individual health care planning.
2. Augmenting resource efficiency through flexibility of access for patients and practitioners using mobile phones and tablets, auto-saving function for ease of use by patients, large data storage capacity and provision of ‘connected information’ for all members of the multidisciplinary team including patients.
3. Strengthening professional accountability through standardisation of needs assessment, promotion of team working and provision of educational audit data.

Based on these premises, ADNAT was included in the UK National Paediatric Diabetes Improvement Plan for 2013–2018 [33]. This inclusion stipulated the need to evaluate ADNAT’s use in clinical practice prior to long-term implementation for which we proposed to follow MRC’s guidance for process evaluation of complex interventions [2] to support its on-going development. This guidance identifies three areas for evaluation which are informed by the causal assumptions of the intervention and interpretation of context, implementation and mechanisms of implementation. This process evaluation model and the theories underpinning the intervention (experiential learning theories and the transtheoretical change cycle [34]) guided the aims of the evaluation study.

## Aims and objectives

### Aims

The aim of this study is to evaluate the feasibility of integrating ADNAT into paediatric diabetes care with respect to:

(i) Resources and processes that influence the clinical implementation of ADNAT
(ii) Methodological issues in preparation for a large scale study

### Feasibility objectives

The objectives of this study are to evaluate:

1. ADNAT’s clinical utility in relation to delivery of paediatric diabetes care
2. How paediatric diabetes health care staff (nurses, doctors, psychologists and dieticians) perceive use of ADNAT within the context of their clinical experiences
3. Key methodological issues that influence sustainability and best practice

## Methods

The objectives were defined more specifically using the RE-AIM framework [35], as recommended in MRC guidance. RE-AIM stands for reach, effectiveness, adoption, implementation and maintenance and included the following:

1. Reach: we assessed the number of participants recruited and retained and response rates to ADNAT, i.e. number completed divided by total number of recruits. Data were obtained from research nurses’ and patients’ ADNAT (monthly) data returns.
2. Effectiveness (potential and perceived): we used NPDA data to assess (pre-study) the functional status of each site, a survey to measure site and practitioners’ views on ADNAT’s effectiveness and collected pre/post-glycated haemoglobin (HbA_1c_) data, taken from patients’ notes by the research nurses, to determine any potential changes in patients’ glycaemic control.
3. Adoption: we carried out a survey to assess system and information quality, accessibility, social norms, data protection and intention of PDUs to use ADNAT in the future. Focus group interviews explored resources needed to set up and sustain use of ADNAT, staff perceptions of factors affecting adoption and their training needs.
4. Implementation/maintenance: the survey and focus groups also explored staff responses to working with ADNAT including perceived value and health improvement outcomes, and the focus group interviews looked at facilitators and barriers to use.

The study was conducted between January 2013 and February 2015. Setup and delivery was supported by the Cheshire and Merseyside Children’s Clinical Research Network (CRN). This support included access to the NIHR CRN-funded research nurses in post at the three NHS Trust sites which were selected based on geography and positive responses to invitation letters. The UK National Research Ethics Service (NRES) defined the study as a service evaluation [36] (see Additional file 1 for the copy of the letter). Approvals from the R&D departments at the three sites were received. The research team had no access to identifiable information for any patient consenting/assenting to participate.

### Participants

The participants were young people with type 1 diabetes aged 12–18 years.

### Respondents, sites and support

Respondents were health professional members of the diabetes teams including paediatric diabetes specialist: nurses, doctors, dieticians and psychologists at three paediatric diabetes centres in the North West of England. These three sites allowed the study to capture diversity of feedback data and ensured adequate representation based on information provided in the 2013–2014 NPDA data (see Table 3). The approach to the implementation of ADNAT was tailored according to team dynamics but each site had a named research nurse for the study, and all members of the team were trained informally by HC to use ADNAT. On-going support was provided by the on-site research nurses and by the ADNAT technologist via email. Each site commenced at a different time point with site 1 starting in March 2013, site 2 in June 2013 and site 3 in February 2014.

### Recruitment

We planned to recruit a minimum of 80 patients attending clinic appointments over an 18-month period. Recruitment was undertaken by the research nurses working in liaison with the diabetes teams to identify young people who met the inclusion/exclusion criteria, as shown in Table 1. A letter of explanation and the study information sheet were posted to eligible young people who were later targeted at their clinic appointments. If they agreed to participate, those under 16 gave signed assent, whilst those over 16 years provided informed consent. Proxy consent of parents/guardians of young people under 16 was also obtained. All information sheets and consent forms were produced in age-and-stage-of-development appropriate formats and were checked before use by an audit team at one of the participating sites. Copies of the signed assent/consent documentation were given to the young people and, where appropriate, their parent/guardian for their records; the original copy was filed in participants’ medical notes, and copies were kept in the site study files held by the research nurses.

**Table 1:** Inclusion and exclusion criteria

Inclusion criteria	Exclusion criteria
Type 1 diabetes (T1D) ≥3 months post diagnosis	Co-existing pathology, e.g. cystic fibrosis
Aged 12–18 years inclusive	In receipt of prescribed medication likely to affect glycaemic control, e.g. systemic steroids
Able to give assent <16 years of age and informed consent >16 years	Have a diagnosed psychological or psychiatric disorder(s) that requires specialist treatment
Have parental/guardian consent for young people <16 years	
Able to complete ADNAT	
Have Internet access at home, school, hospital, public library or via mobile technology	

### Delivery of ADNAT

Participants were provided by the research nurses with username and password access to the ADNAT App, alongside their usual standard care based on the BPT [8] criteria (3-monthly follow-up including HbA_1c_, and tailored self-care education; annual review of body mass index, blood pressure check and screening for eye and kidney problems from age 12, plus psychological assessment). The ADNAT App was accessed through the Internet using a PC, laptop or mobile technological devices including participants’ mobile phones or tablets. They could choose where to complete it: at home and/or in clinic on their own smart phones or using iPads loaned to them by the research nurses. All participants were followed up at their diabetes outpatient clinics or at a home visit. In both cases, the ADNAT outcome data was used to guide their health care plans. Those who successfully completed and submitted their ADNAT questionnaires were called the ‘completers’, whilst those who chose not to submit were called the ‘non-completers’ and were used as the comparative group.

### Quantitative feasibility outcome data

A range of feasibility outcomes were measured including:

- ADNAT data to measure reach in terms of response rates across the PDUs. All data, which was automatically downloaded onto a secure central database, were encrypted to ensure anonymity. Data included number of participants recruited and retained and response rates to ADNAT and ADNAT NARs for self-care and psycho-social health.
- National Paediatric Diabetes Audit (NPDA) data [4] to assess the functional status (effectiveness) of each site.
- Glycaemic control to compare potential effectiveness pre/post-intervention levels of glycaemic control using baseline HbA_1c_ levels (means/standard deviations over previous 12 months), and 6-month post-ADNAT levels obtained from patients’ clinical notes by the research nurses.
- A 67-item survey to collect information on perceived effectiveness, adoption, implementation and maintenance. Adapted from a validated survey developed by Okazaki et al [37], it has seven domains including system and information quality, accessibility, perceived value, data protection, health improvement, subjective norms and intention to use in the future. We also included an open-ended question at the end asking if there was anything they would change. The survey was facilitated by the Audit Department at one of the participating sites using SNAP software (http://www.snapsurveys.com/) and was pilot tested by two researchers. It was sent out by the Audit Department via an email link to all respondents. Responses were returned directly to the Audit Department where analysis of the data was completed using SNAP software.

### Qualitative process evaluation data

Three focus groups were run at the end of the study period, one at each of the three sites. All respondents were invited to participate, and they were sent information sheets and an interview schedule prior to the meetings. The schedule was informed by the RE-AIM domains. It aimed to evaluate training needs, staff perceptions of facilitators and barriers to using ADNAT, and staff responses to working with ADNAT including perceived value and perceived health improvement outcomes. Consent forms, prior to participation, were signed for tape-recording the interviews.

### Progression criteria

We specified a priori progression criteria that should be met in order for continuation to the main study. These included the following: that recruitment to the study should be ≥30% of the PDUs’ eligible 12–18 years populations attending clinic, that there should be no deterioration in mean HbA1c levels at 6 months in participants and that diabetes teams should report positive feedback on ADNAT’s perceived effectiveness including system and information quality and accessibility and be tailoring ADNAT to meet their site needs.

### Data analysis

The encrypted quantitative ADNAT data were collated, coded and analysed using R or SPSS. All quantitative data taken from the ADNAT questionnaires were checked for missing or unusual values and for internal consistency of the scoring items. Participant glycaemic control (HbA_1C_) was monitored pre and post use of ADNAT at each site and across all sites using summary statistics for completers and non-completers. Analysis of covariance was performed with post-HbA_1C_ as the dependent variable and baseline HbA_1C_, completer status and site as the independent variables to assess whether any preliminary change in HbA_1C_ levels was apparent. Correlations between high/moderate self-care needs (based on the self-care NAR) and poor/moderate levels of (pre-baseline and at 6 months) HbA_1c_ were analysed using Spearman’s rho statistic [38].

Qualitative data were analysed using an inductive thematic content analysis, assisted by *QSR NVivo* software. First, an evolving set of themes was created and linked to respondents’ ‘quotes’. These themes were then categorised within the RE-AIM domains. To assure trustworthiness of the analysis, respondent validation was used by cross-checking findings with respondents and triangulating it with the quantitative outcomes.

## Results

Data from recruitment of patients, the NPDA, the survey and focus groups comprise the results of the study. The RE-AIM domains are used to present both the quantitative and the qualitative data.

### Comparison of ADNAT completers versus non-completers

#### Reach

Table 2 shows recruitment rates and participant characteristic data. We planned to recruit a minimum of 80 patients and we recruited 89 in total, with an uptake of 65–70% of those who were screened as eligible to participate. The graph in Additional file 2 shows that actual recruitment rates were above our monthly anticipated target rates. Of those recruited, there were twice as many females to males and the withdrawal rate was low at 4.5% (*n* = 4). Reasons for withdrawal included patient transfer to other areas (*n* = 1) and not wanting to continue with the study (*n* = 3). Forty-four young people (49.4%) submitted their completed ADNAT questionnaires. There were more female than male completers and non-completers (ratio ~1 male to 2 females), and their average age was 14.3 years compared to 14.5 years for the non-completers. Forty-eight of the young people were aged 11–14 years (25 completers, 23 non-completers), and 41 were aged between 15 and 18 years (19 completers, 22 non-completers).

**Table 2:** Participant (patient) characteristics

Site	1	2	3	Combined
No. recruited	28	26	35	89
Male/female ratio	8:20	10:16	12:23	30:59
Withdrawals (%)	0	2 (7.7%)	2 (5.7%)	4 (4.5%)
ADNAT completers				
*N* (%^a^)	13 (46.4%)	18 (69.2%)	13 (37.1%)	44 (49.4%)
Male/female ratio	4:9	8:10	3:10	15:29
Mean age, years (range)	14.3 (12–16)	14.4 (12–17)	14.3 (12–16)	14.3
ADNAT non-completers				
*N* (%^a^)	15 (53.6%)	8 (30.8%)	22 (62.9%)	45 (50.6%)
Male/female ratio	4:11	2:6	9:13	1:2
Mean age, years (range)	14.7 (11–17)	15.3 (12–18)	14.1 (12–17)	14.5

^a^Percentage of the number recruited

#### Effectiveness (potential): glycaemic control

Data in Table 3 are taken from the 2013/14 NPDA [4]. It shows disparity between the sites for the percentage of young people who have a mean HbA_1c_ of the recommended level of less than 58 mmol/mol (range 8.1–26.5%), with the mean (range 65.5–78.7 mmol/mol) and median values (range 64–74 mmol/mol) for all sites above the recommended level. The NPDA ascribes such variability (despite statistical adjustments for known confounding influences, such as ethnicity, social deprivation, gender, age and diabetes duration) to differences in service provision and delivery which has particular relevance for this study. In relation to this, care process records, which are used to monitor diabetes management and detect long-term complications at the earliest treatable stage, were also significantly different in terms of incomplete records (range 25.4–69.1%), again highlighting disparity between the three sites. Of note is the fact that the two with poorer HbA1c audit results (sites 1 and 3) had interruptions in team functioning during the study period due to staff changes and/or long-term staff absences owing to sickness.

**Table 3:** Summary outcome data for the three study sites taken from the 2013–2014 National Paediatric Diabetes Audit

Site	1	2	3
Total number of patients (aged 10–18 years)	248 (211)	110 (98)	121 (99)
HbA_1c_ <58 mmol/mol (normal HbA1c range = 20–41 mmol/mol)	16.6%	26.5%	8.1%
Mean HbA_1c_	72.4	65.5	78.7
Median HbA_1c_	69.0	64	74.0
% incomplete records of care processes (except HbA_1c_)	25.4%	40.7%	69.1%

**Fig. 1:**
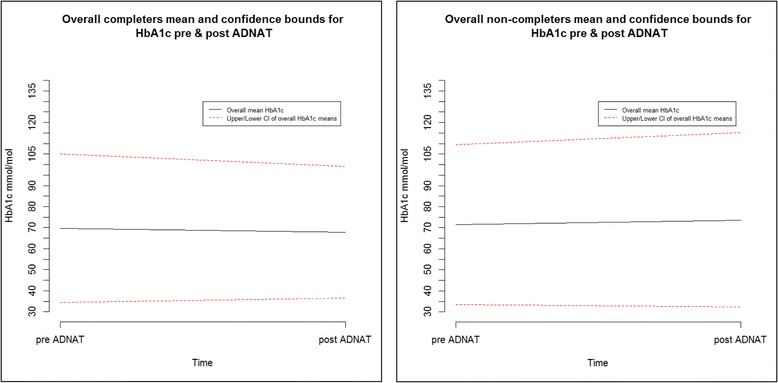
Plot of pre- and post-mean HbA_1C_ levels for completers and non-completers

Table 4 shows the glycaemic control data pre- and 6 months post-ADNAT for the completers versus the non-completers. For both groups, subject-specific profile plots (not shown) and the range of pre- and post-HbA_1C_ levels indicated that the young people had very different pre-glucose levels and variable rates of change leading to their post-HbA_1C_ levels. Overall, summarising across all three sites, there was a non-significant reduction in the post-ADNAT mean and median HbA_1c_ levels for the completers versus a non-significant increase in the mean and median levels for the non-completers. The mean HbA_1C_ levels are illustrated in Fig. 1 and suggest a potential decreasing trend in HbA_1C_ for ADNAT completers. This trend is encouraging given that our progression criteria defined ‘no deterioration in HbA_1c_’ as the outcome at 6 months.

**Table 4:** Participant (patient) glycaemic control data pre/post-ADNAT

Summary measure	Site 1	Site 2	Site 3	All sites
Completers				
Pre-mean HbA_1c_ mmol/mol (sd)	73.1(22.4)	64.8(15.9)	74.6(14.8)	70.2(18.0)
Post-mean HbA_1_c (mmol/mol) (sd)	63.1(12.6)	64.7(9.8)	75.9(21.7)	67.7(16.0)
Number of pre ADNAT	13	18	13	44
Non-completers				
Pre-mean HbA_1c_ mmol/mol (sd)	78.9(21.0)	61.9(13.2)	68.8(18.8)	71.0(19.4)
Post-mean HbA_1c_ (mmol/mol) (sd)	81.4(23.6)	63.7(13.2)	71.0(20.5)	73.4(21.3)
Number of pre ADNAT	15	8	22	45

*sd* stands for standard deviation

The results of the analysis of covariance (ANCOVA), presented in Table 5, show how post-ADNAT mean HbA_1C_ levels changed after adjustment for pre-HbA_1C_, completer status and site. In general, the model explained a reasonable amount of the overall variability in post-HbA_1C_ levels with a *R* ^2^ value of 0.52. Only pre-HbA_1C_ mean level was a strong predictor of post-HbA_1C_ mean level (*p* < 0.001), which is to be expected since the two measures are correlated. On average, post-HbA_1C_ levels increased by 0.71 mmol/mol for each unit increase in baseline HbA_1C_. The completer status variable reached borderline significance (-5.42, 95% confidence interval −11.48, 0.64), indicating that on average, completers had a post-ADNAT mean HbA_1C_ level of 5.42 mmol/mol lower than non-completers. Mean differences between site 2 and site 3 compared to site 1 indicated a lower average post-HbA_1C_ mean difference by 1.75 mmol/mol at site 2, and a higher average mean difference by 1.50 mmol/mol at site 3, compared to site 1, after adjusting for baseline HbA_1C_ and completer status, but these differences were non-significant. Please note that the results from the above model should be interpreted with caution due to the small numbers at each site.

**Table 5:** ANCOVA regression analysis on post-HbA_1C_ levels

Variable^a^	Estimate	Std. error	95% confidence interval
Intercept	22.79	6.31	10.42, 35.16
Pre-HbA1c	0.71	0.08	0.55, 0.87
Completers	−5.42	3.09	−11.48, 0.64
Site 2	−1.75	4.11	−9.80, 6.30
Site 3	1.50	3.92	−6.18, 9.18

^a^Reference categories are non-completers and site 1

**Fig. 2:**
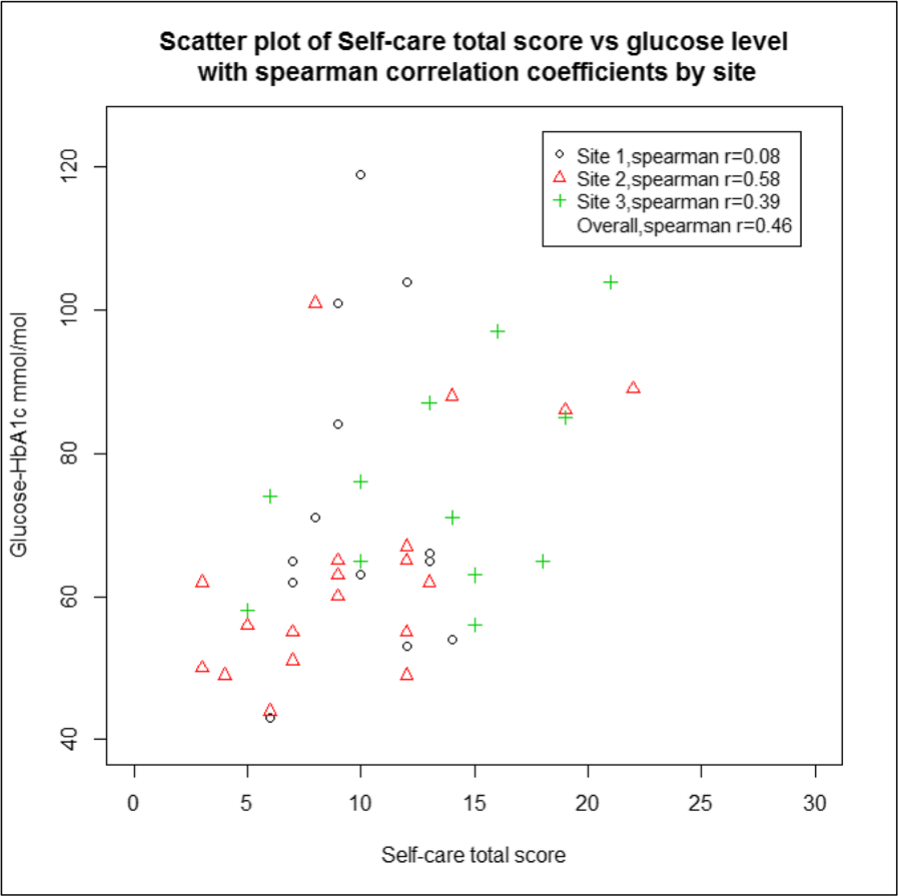
Scatterplot of HbA_1C_ level and self-care total score at 6 months

Figure 2 shows a scatter plot of the correlation between HbA_1C_ levels and self-care scores. The Spearman rho coefficient is 0.46 suggesting a good moderate correlation with higher (worse) self-care scores indicating higher HbA_1C_ levels overall. Only at one site was very little correlation observed due to several outlying young people with high HbA_1C_ levels but generally lower self-care scores.

All questions comprising the self-care score are listed in Table 6 together with the number of children and percentage scoring green, amber and red at each site using the ADNAT scoring algorithm. Examples of the response categories, comprising green, amber and red coding, are also given for a selection of the items. The table indicates that a large proportion of young people were scoring green for each item, indicating reasonable management, but box plots by item and site (not shown) suggested that there was variability across items and sites and that it was not necessarily the same group of young people scoring green across all items.

**Table 6:** Summary of item scoring classifications of 20 ADNAT self-care questions with examples of some of the shorter range of responses (in brackets)

			Scoring algorithm summaries count (%)		
Domain	Item no.	Question (responses comprising green/amber/red)	Green	Amber	Red
Physical activity	16	How many hours of pulse-raising exercise or physical activity did you do last week? (7-8 hours or more than 8 hours/3-4 hours or 5-6 hours/Less than 1 hour or 1-2 hours)	8 (18%)	19 (42%)	18 (40%)
	18	What stops or prevents you from starting to do exercise or physical activity?	26 (58%)	2 (4%)	17 (38%)
	21	What makes it difficult to manage your blood glucose levels when exercising or doing physical activity?	19 (42%)	9 (20%)	17 (38%)
	22	What usually happens to your blood glucose levels when you do exercise or physical activity?	23 (51%)	15 (33%)	7 (16%)
Eating	34	Do you eat fruit and/or vegetables? (Yes usually every day/Sometimes but not every day/No or very rarely)	28 (62%)	16 (36%)	1 (2%)
	35	How many times a week do you eat treats, such as sweets, chocolate, fast food, takeaways? (1-2 or Less than once per week/3-4 days/Every day or 5-6 days)	20 (44%)	15 (33%)	10 (22%)
	38	Which statement best describes you? (diet control)	34 (76%)	11 (24%)	0 (0%)
	41	Which statement most applies to you? (carbohydrate calculation)	42 (93%)	0 (0%)	3 (7%)
	45	Are you happy with your weight? (Happy with weight and never tried to gain or lose/Happy with weight but have tried to gain or lose/Not happy with weight or Prefer not to say)	19 (42%)	12 (27%)	14 (31%)
Monitoring blood glucose	50	How often do you normally test your blood glucose in a day? (Once or twice or 3-5 times or More than 5 times/It varies a lot depending on where I am or what I’m doing/I don’t usually test my blood at all)	35 (78%)	10 (22%)	0 (0%)
	51	What motivates you to test your blood glucose?	40 (89%)	5 (11%)	0 (0%)
	53	How would you describe your blood glucose results? (They are generally within the recommended levels/They always go up and down/They are usually high or They are usually low or I’m not sure or I don’t know because I don’t usually test my blood)	20 (44%)	21 (4%)	4 (9%)
	57/62	How often do you have hypos/low blood glucose (less than 4 mmols/l)? Have you been unconscious from hypoglycaemia in the last 12 months?	6 (13%)	33 (73%)	6 (13%)
	66/71	How often do you have high blood glucose (more than 10 mmols/l)? Have you had diabetic ketoacidosis in the last 12 months (not including at diagnosis)?	24 (53%)	5 (11%)	16 (36%)
	76	What would you like your HbA1c to be?	39 (87%)	0 (0%)	6 (13%)
Medication taking	80	What motivates you to do your injections or to give insulin through your pump?	30 (67%)	14 (31%)	1 (2%)
Living with diabetes	92	What would you do if you were ill with an infection (e.g. sickness/flu) and it made your blood glucose high?	38 (84%)	1 (2%)	6 (13%)
	94	You are staying over at your friend’s house. Which of the following would you do?	42 (93%)	1 (2%)	2 (4%)
	111	You are going to a party one Friday night with your friends and you know that they will be drinking alcohol. Which statement best describes what you would do?	37 (84%)	4 (9%)	3 (7%)
	112	Which statement best describes you? (diabetes and life)	13 (30%)	24 (54%)	7 (16%)

### Survey

Eleven people (two males, nine females) out of 14 completed and submitted the survey. They included seven nurses, one doctor, two dieticians and one research nurse. When asked about years worked in paediatrics, two had worked for five or less years, two for 6–10 years, five for 11–20 years and two for 21–25 years. Two people had a recognised adult teaching certificate (English National Board 998 Adult Teaching and Assessing course), but no one had a paediatric teaching qualification.

#### Adoption

Table 7 shows responses to statements relating to adoption of ADNAT. Given the ad hoc nature of the scales used, single item responses are reported (as opposed to scale-scores) which cover the following areas:

**Table 7:** Responses to statements concerning system and information quality and intention to use ADNAT in the future

Information quality: the information I obtained from ADNAT	Yes	No	Not sure			
Was easy to understand	9		2			
Was easy to interpret	8	1	2			
Included all necessary assessments	9		2			
Was sufficiently complete to meet my patients’ needs	7		4			
Had sufficient breadth and depth for my patients	8	1	2			
Total	41	2	12			
System quality	Strongly disagree	Disagree	Neither agree nor disagree	Agree	Strongly agree	Not applicable
ADNAT is easy to use			1	6	3	1
ADNAT is equipped with useful features and functions			1	7	2	1
ADNAT is easy to complete				6	3	2
ADNAT is always available to use		1	3	5	1	1
ADNAT launches and runs right away		2	5	2	1	1
ADNAT does not crash		1	5	3	1	1
ADNAT does not freeze after entering or retrieving information		1	4	4	1	1
The commands of ADNAT are well depicted by symbols and buttons			3	5	2	1
The layout of ADNAT is clear and consistent			1	6	3	1
The design of ADNAT is easy to use or operate		1	1	5	3	1
The Technologist showed a sincere interest in solving my problems			2	3	1	5
The Technologist gave me personal attention			3	2	1	5
The Technologist was dependable			3	2	1	5
Total		6	32	56	23	26
Intention to use in the future						
Technology is an important element of my patients’ education			2	3	6	
Without Technology I would be unable to do my work			3	2	6	
Technology makes my work more enjoyable		1	7	1	2	
My workplace is not good in the way it uses Technology	1	2	4	2	2	
With Technology I interact more with my patients		3	2	4	2	
I find using computers difficult	6	3	2			
I find using technological devices difficult e.g. mobile phones, iPads	6	3	2			
Getting access to Wifi is a problem in my work place	1	1	2	7		
Technology makes my work easier			4	5	2	
It would be good if Technology was used more			4	4	3	
Total	14	13	32	28	23	0

- *Information and system quality*: the majority of respondents (*n* = 7–9) gave a positive response to the five statements relating to information quality, suggesting confidence in ADNAT. However, there was uncertainty for four participants in relation to ADNAT being sufficiently complete to meet their patients’ needs. Overall, the majority of participants strongly agreed or agreed with the 13 statements relating to system quality, but some ambivalence was expressed in relation to ADNAT launching and running quickly and not crashing.
- *Intention to use ADNAT in the future, accessibility and capability with technology*: ten respondents said they intended to use ADNAT when it is available at their work place. However, the majority (*n* = 9) indicated that accessing Wi-Fi is a problem in their work place, and four people felt that their workplace is not good in the way it uses technology. Nine reported that patients had completed ADNAT at home and five in clinic. All respondents felt that ADNAT was secure with regard to data protection. When asked about using technology in clinical practice, nine respondents reported capability with computers, tablets and mobile devices; the remaining two were ambivalent. Nine people regarded technology to be an important element of their patients’ education.
- *Social norms* (not shown in Table 7): when asked to give an opinion on whether their National Children and Young Peoples’ Diabetes Network, their managers, their colleagues, and their patients and families would think they should be using ADNAT, all participants (*n* = 11) responded positively to the Network and patients and families options, and seven positively responded to the managers and colleagues options.

#### Implementation/maintenance

Table 8 summarises survey responses to questions relating to implementation and maintenance of ADNAT which included the following:

**Table 8:** Responses to statements concerning perceived value of ADNAT and health improvement outcomes

Statements	Poor	Fair	Average	Good	Excellent	
Perceived value of ADNAT in relation to						
Effectiveness		1	2	8		
Practicality		2	4	5		
Usefulness			1	8	2	
Efficiency			4	6	1	
Total		3	11	27	3	
The value placed on ADNAT by my patients depended upon						
	Yes	No	Not sure			
Age	6	1	4			
Gender		4	7			
Reading and numeracy skills	6		5			
Previous diabetes education	6	1	4			
Parental support	8		3			
Insulin regimen		4	7			
Hospital admissions		4	7			
No. of contacts with diabetes team	3	2	6			
How frequently did ADNAT help your patients in relation to						
	Never	Seldom	Sometimes	Often	Regularly	Not applicable
Enlisting help		3	6	1	1	
Increasing knowledge about managing diabetes		1	6	2	1	1
Being aware of personal risks		1	8	0	1	1
Understanding benefits of changing behaviour(s)	1		8		1	1
Committing to changing behaviour(s)		1	8		1	1
Developing a plan for changing behaviour(s)	1	1	8		1	
Changing behaviour(s)		1	8		1	1
Being aware of relapse	1	1	6	1	1	1
Total	3	9	58	4	8	6

- *Perceived value*: overall, respondents judged ADNAT to be effective, practical, useful and efficient, with nobody judging it to be poor. In relation to factors that influenced its value to patients, respondents were unsure about gender, insulin regimen and hospital admissions, but more confident with regard to parental support, age and previous diabetes education.
- *Health improvement outcomes*: statements here were based on the transtheoretical change cycle. The majority of participants indicated that ADNAT had an effect at each of the eight different stages of the cycle with the majority indicating that effects happened sometimes, often or regularly. The ‘not applicable’ responses came from the research nurse who was not involved at the clinical level and were therefore not included in the calculations.

When asked to add any comments about what they would change about ADNAT (not included in Table 8), five responses were received including the need for iPads and improved Wi-Fi access in clinics, access to on-line reports and inclusion of a section for patients to ask for immediate feedback/help from the diabetes team.

### Focus groups

Each site and participant was coded (based on roles and numbers) for reference purposes as follows: Paediatric Diabetes Specialist Nurses (PDSN 1–6), Doctors (Dr 1,2), Researcher (R 1,2), Psychologist (P) and Dietician (Di). Twelve people in total attended the three groups (site 1: *n* = 5, site 2: *n* = 3, site 3: *n* = 4) providing a total of 160 min of recorded conversation. Analysis of the focus group data produced seven sub-themes which were aligned to the RE-AIM framework (themes). Findings are presented using anonymised quotes to capture the essence of the phenomena and are summarised at the end in Tables 9 and 10 which provide a combined summary of the quantitative and qualitative data and a synopsis of practice recommendations.

**Table 9:** Summary of quantitative and qualitative data for feasibility outcomes

RE-AIM themes	Outcomes (patients)	Survey (diabetes team)	Focus groups (diabetes team)
Reach	• Uptake better than expected (*n* = 89) • Twice as many females to males recruited • Low accrual rates (*n* = 4) • Response rates 49.4% • Average age of completers and non-completers 14.3/14.5 years respectively • More female than male completers: ratio ~2:1	• All reported technological capability in clinical practice • Some ambivalence re: using technology in patients’ education • No paediatric teaching qualifications	• Ideal time to integrate ADNAT into clinical practice • Offers a technological approach to care in line with policy and young peoples’ needs • Fits within BPT’s education criterion/peer review process. • Potential as an audit tool questioned given its focus on 12–18 years only • Training to use activity learning to support a team approach and include expert users of ADNAT
Effectiveness (potential and perceived)	• Completers—post-ADNAT mean HbA_1C_ level 5.42 mmol/mol’s lower than non-completers at 6 months • ADNAT judged to be effective at each of the 8 different stages of the transtheoretical change cycle	• ADNAT’s system and information quality judged as good • ADNAT judged to be effective, practical, useful and efficient • Value to patients perceived to be linked to parental support, age and previous diabetes education	• Time between patients’ completions and reviews with practitioners in clinic/home critical to effectiveness • ADNAT perceived to promote behaviour change • Primary outcomes to include glycaemic control and quality of life, with qualitative data to illuminate wider effects of education
Adoption		• Majority of patients completed ADNAT in clinic • All felt that patients, their families and the Diabetes Network would want ADNAT to be used • Majority intend to use ADNAT in the future	• Lead clinician support essential • Requires a team approach to implementation • Needs to be tailored to fit each team • Scoring, traffic light feedback, drop-down menus and navigation commands support tailored health care planning but some concern re: patients’ responses to traffic light system
Implementation/maintenance		• Access to Wi-Fi in clinics poor/negligible • No data protection issues reported • Need for: – Improved Wi-Fi access and IPads – Section for patients to ask for immediate feedback/help from diabetes teams – Access to short on-line patient reports	• Access to on-line technical support needed • Use of Ipads with SIM cards to overcome Wi-Fi problems in clinics • To secure clinical feasibility: – Home completions prior to clinic visits – Consents taken in clinic – Instructions sent in appointment letters – Automatic text reminders

**Table 10:** Practice implications

Reach	• Lead clinician’s support required alongside a team approach to foster integration, normalisation and consistency in the messages given to patients and their carers
Effectiveness (potential and perceived)	• Time between patients’ completions and clinic reviews critical to success
Adoption	• Access to technical support and iPads with SIM cards to overcome Wi-Fi problems in clinics • Staff training: use an activity style of learning and limit to a maximum of 4 h to support team working and the tailoring of ADNAT for each team • Include expert patients in the training to provide insights into their ADNAT experiences to support connections between theory and practice
Implementation/maintenance:	• Consents to be taken in clinic, followed by instructions at a later date in patients’ appointment letters • ADNAT to be completed at home prior to their clinic consultation, supported by automatic text reminders • Short time span needed between patients’ completions of ADNAT and follow-up reviews ADNAT requires: • Section for patients to ask for immediate feedback/help from diabetes teams • Short on-line reports, i.e. ADNAT profiles for each patient for practitioners • Less threatening feedback response for patients to overcome potential negative responses to the (red) traffic light feedback system

#### Adoption themes

- Tension for change

Respondents found ADNAT to be a viable option for clinical practice and wanted to change the way they engaged with their patients by using technology, recognising that web-based applications play a crucial role in adolescent life. They perceived technology as a way of overcoming communication barriers, as this nurse commented,

I remember being quite impressed at what they were saying they didn’t know. So it seems to get past that barrier when it is face to face. They are more likely to be honest even though they know we’re going to see it (PDSN4).

- System fit

The three sites all felt that ADNAT fit within their teams’ values and goals, with participants suggesting that it could standardise educational assessment allowing for comparisons between PDUs. As a policy driver, the BPT enhanced motivation to use ADNAT, linking it to the education criteria. Other respondents agreed with this thinking suggesting that it also met with the peer review process but questioned its practical potential as an audit tool given that it assesses those aged 12–18 years only making it ‘difficult to draw any conclusions’ (Dr1).

- Organisational working

Operationally, decision-making regarding how to implement ADNAT was devolved to the teams to see what emerged. Two different methods were used: individual nurses reviewing their own patient returns versus using a generic email for all returned questionnaires which was reviewed by one PDSN only. For the former, choice of treatment was determined by the individual nurses but they also discussed their approaches within their teams. For the latter, the PDSN identified urgent cases, i.e. red traffic light returns for discussion at team meetings. Both approaches therefore embraced working as a team, as the following quote highlights,

We did bring the red ones to the team meeting and there were actions..generated from it, and we did implement those actions. I think the ones that came through green reinforced what we felt but it was good to get the teenagers’ perspectives married up with ours (PDSN2).

- Team working

Team capacity varied owing to sickness absence and/or new staff starting. Site 2 was not affected by these problems and had an established team. It was notable that this site had the best 2013/2014 audit returns in relation to glycaemic control (as highlighted in Table 3) and also the best ADNAT return rates (69.2%), compared with the other two sites (46.4 and 37.1%). With regard to team working, participants commented on how ADNAT encouraged a standardised approach which supported consistency in the messages given to patients. They argued that integrating ADNAT into their team work would normalise its use, although lack of time, given the current politically driven changes, had impacted upon feelings of being in control. Respondents talked about ‘time constraints’, ‘feeling too busy’ and ‘a continual focus on problems’. Receptivity for change therefore varied across the sites although respondents felt that ADNAT had the potential to drive change.

#### Implementation/maintenance themes

- Time

The time taken for patients to complete ADNAT was discussed given the large number of questions to be answered, but two respondents had asked patients for their perspectives and both reported positive responses,

I asked quite a few of them, was it a waste of 30 minutes of their life and they all said ‘no’ they felt it was useful that they’d done it, and many of them said it made them think… (Dr2)

..a lot of them came back and said it was a good use of their time and gave them that refreshment of the advice that we gave them previously (PDSN6).

For the practitioners, the time taken to review patients’ outcome results was helped by ADNAT’s scoring and traffic light feedback systems, alongside the drop-down menus and navigation commands to allow selection of scoring questions only and/or questions relating to the different domains. However, when asked whether they felt the traffic light system was good for the children, there was a mixed response. It was viewed as both a facilitator and a barrier, with the barrier relating to its potential to raise young peoples’ anxiety.

- Embedding ADNAT into practice

There were mixed responses with regard to where ADNAT should be completed. Location was seen as important affecting both uptake and practitioner feedback to patients. Home completions brought problems in relation to patients being willing to complete it once they left clinic, and time between completion and feedback was deemed important, as the following quote highlights,

Because they did it at home and sometimes then a week after their previous clinic appointment, then it would be reviewing it again much later. …and actually they couldn’t remember the results (PDSN4)

Theoretically, completions in clinic prior to their consultation were thought beneficial, but practically, this was not an option given time limitations. Home visits were appropriate for two of the sites, but at the third site, home visits were being discouraged by management. These comments highlighted a barrier to embedding ADNAT into practice. When questioned about how this could be overcome, integrating ADNAT into patients’ health care plans was seen as a viable option, with patients completing ADNAT prior to their next clinic appointment at home. Suggestions included gaining consents in clinic and sending instructions on how to complete ADNAT with their clinic appointment letters, followed by text reminders. The role of the lead clinician was seen as crucial for embedding ADNAT into routine practice, alongside mandating its use through, for example, including it in the BPT criteria. Tailoring ADNAT to fit each site was seen as important, paying attention to the whole team being involved. To meet this goal, training (up to a maximum of 4 h) was considered essential. Web-based instruction was not popular given the need for self-motivation and personal time, but face-to-face training was deemed superior in that it would,

..help to promote the team aspect of it because discussion could be had about how to make it cohesive as a team (PDSN4).

Another suggestion was to include previous users of ADNAT, i.e. expert patients in the training programme.

- Linkages

Respondents felt that ADNAT mapped on to what they are aiming to achieve in clinic including getting patients to “create (their own) agendas and identify things” (P). Other respondents felt that it provided the link between all the different components of diabetes self-management commenting that ADNAT got patients “thinking about aspects of the condition which they might normally not really think about” (PDSN 3). There were comments that in clinic, the focus tends to be on blood glucose and insulin doses whilst ADNAT promoted reflection on all aspects of their diabetes, including their feelings. One person summarised ADNAT’s perceived value in the following way,

the opportunity for self-evaluation of learning, reflection, and for young people to actually get feedback on what they know, and also for the teams to have feedback on what they know as young people (Di).

This process of self-evaluation was a strong theme throughout the focus groups with one nurse commenting that ADNAT, “…reminded them (patients) about the right ways to manage their diabetes” (PDSN6). There was an agreement that ADNAT promotes behaviour change, and in terms of why it is effective, one person summarised her opinion by saying that,

It gives them (patients) a chance to identify. They’re doing the identifying, possibly prioritising things for themselves …and if it has come from them, then they are much more likely to engage in conversations about what could be done differently…. (P)

Respondents questioned the traditional (misplaced) focus on glycaemic control with one person stating that education is more about quality of life at this age and being able to ‘… get a balance between their diabetes and being a teenager…’ (PDSN4). This point was agreed by others who felt that a single educational intervention is not going to impact upon glycaemic control because there are ‘an awful lot of things that affect someone’s HbA_1c_’ (Dr.2). Education was seen as beneficial in other ways including improving quality of life and self-care processes, and the example of carrying glucose to treat hypoglycaemia was used to highlight this point.

Having open-ended text responses at the end of each question was seen as important, because it allowed patients to express their feelings of knowing more and being in control. This concept of ‘control’ was an important theme, with ADNAT being viewed as a way of accessing patients’ needs without removing their sense of control, as the following quote highlights,

..it might be a question that they (patients) might not have thought about, but felt a bit embarrassed, or thought well, I shouldn’t think like that, or maybe other people don’t feel or think like that, I should know that. (PDSN3)

Accessing patients’ needs meant that the teams could tailor conversations with their patients, focusing on their raised self-awareness with regard to what they did and did not know, providing a base on which to progress joint health care planning.

#### Combining the data

We used the RE-AIM framework to combine the data from recruitment of patients, the NPDA, the survey and the focus groups. This summary of the quantitative results and the qualitative findings is provided in Table 9. We also identified the main points raised for how best to integrate ADNAT into clinical practice (summarised in Table 10).

## Discussion

This study aimed to evaluate the feasibility of integrating ADNAT into paediatric diabetes care focusing primarily upon resources and processes that influenced its implementation taking into account context and perceived impact. We met each of our progression criteria, recruiting over 65–70% of those screened as eligible to take part; there was no deterioration in mean HbA1c levels at 6 months; and diabetes teams reported positive feedback on ADNAT’s perceived effectiveness and tailored the use of ADNAT within their clinics to meet their site needs.

The study also aimed to determine methodological recommendations for a future large-scale study. Its strength lies in its mixed methods design and the fact that there was overlap between the different data sets which supports the findings and helps to explain the feasibility outcomes. The summary of the quantitative results and the qualitative findings in Table 9 shows that from a quantitative (reach and potential and perceived effectiveness) outcome perspective, ADNAT met the proposed mechanisms of action and progression criteria from a staff perspective. The survey and qualitative findings indicate that ADNAT was acceptable to the diabetes teams. However, these results need to be interpreted with caution due to the study design and the associated confounding factors.

## Methodological implications: strengths and limitations

A limitation of this study is the small number of sites, participants and respondents involved, alongside the non-randomisation to treatment. Both of these limitations raise statistical issues concerning the accuracy of the outcome data. Differences in characteristics between those patients who chose to complete ADNAT compared to the non-completers are unknown, and it can be argued that the former group may be more compliant generally with regard to their diabetes self-care when compared to the latter group. However, this potential difference was accounted for by controlling for HbA1c levels at baseline. Findings therefore provided an insight into ADNAT’s potential in relation to glycaemic control (HbA1c), particularly for those poorly controlled. However, it can also be argued that those young people who completed ADNAT may have wanted a different kind of management for their diabetes, one that fits more effectively with a digital culture and their learning styles. This suggests that completers may have been ready to make changes compared to the non-completers. Reasons why young people choose to engage or not in using ADNAT therefore need to be researched to improve future response rates.

Questions regarding the number of young people who completed ADNAT also need to be addressed. Whilst the percentage of completers (49.4%) is good in terms of figures quoted for the response rate of the general population to web-based surveys (24.8%) [39], it remains questionable as to how typical the completers were relative to the non-completers and to those who declined to participate. It highlights the need to identify ways of engaging more effectively with what is typically a ‘hard to reach’ population. Systematic reviews [40, 41] suggest extended timeframes, recruitment techniques suited to young people and the need to work in close co-operation with the community.

The study identified the research setting as a mediator of outcomes making treatment heterogeneity a confounding factor. Whilst ADNAT is a standardised intervention, the responses of the teams to the outcomes of using ADNAT are heterogeneous. This demonstrated a need to include non-participant clinic observations in a future study to evaluate what is actually happening in clinical practice when ADNAT is used. It also validated the need to research comparative effectiveness of the different research sites by using the historical NPDA data to provide evidence on the value of different response options to ADNAT.

Previous research has highlighted the need for adult diabetes education to have broad patient-based outcomes and not to be expected to have lasting benefits on glycaemic control unless it is repeated. The focus group data reinforced these points highlighting the need to include other outcomes such as quality of life and patients’ greater self-involvement in their care. Findings from previous trials of paediatric diabetes education have also affirmed this point [15]. Measures of effectiveness therefore need to include but not be limited to glycaemic control.

## Conclusions

This evaluation study has provided evidence on the feasibility of using ADNAT in clinical practice and has demonstrated a number of limitations which have provided practice and methodological guidance. It has shown that a randomised design that fits the needs of a ‘hard to reach’ adolescent population is necessary. A cluster randomised controlled trial that involves sequential but random rollout of ADNAT over multiple time periods may the most appropriate and is currently being considered for the larger study.

## Additional files

Additional file 1: Ethics. (PDF 169 kb)

Additional file 2: Recruitment. (DOCX 14 kb)

## Abbreviations

ADNAT: Adolescent Diabetes Needs Assessment Tool
ANCOVA: Analysis of covariance
BPT: Best Practice Tariff
CI: Confidence interval
CRN: Clinical Research Network
MRC: Medical Research Council
NAR: Needs Assessment Rating
NHS: National Health Service
NIHR: National Institute for Health Research
NPDA: National Paediatric Diabetes Audit
NRES: National Research Ethics Service
PDU: Paediatric Diabetes Unit
R&D: Research and Development
RE-AIM: Reach, effectiveness, adoption, implementation, maintenance
T1D: Type 1 diabetes
TEL: Technology enhanced learning
