# Supplementary material for: A mixed methods study to evaluate the feasibility of using the Adolescent Diabetes Needs Assessment Tool App in paediatric diabetes care in preparation for a longitudinal cohort study
Source: Pilot Feasibility Stud. 2017 Jul 6;4:13. doi: 10.1186/s40814-017-0164-5 (PMC5501574; doi:10.1186/s40814-017-0164-5)
Supplement: Supplementary file 2 — Recruitment. (DOCX 14 kb) [file 40814_2017_164_MOESM2_ESM.docx]

**Additional file 2**

Figure 1: Recruitment rates: actual set against target rate (March 2013 - September 2014)
