# Supplementary material for: A mixed methods study to evaluate the feasibility of using the Adolescent Diabetes Needs Assessment Tool App in paediatric diabetes care in preparation for a longitudinal cohort study
Source: Pilot Feasibility Stud. 2017 Jul 6;4:13. doi: 10.1186/s40814-017-0164-5 (PMC5501574; doi:10.1186/s40814-017-0164-5)
Supplement: Supplementary file 1 — Ethics. (PDF 169 kb) [file 40814_2017_164_MOESM1_ESM.pdf]

NHS
Health Research Authority
National Research Ethics Service

NRES Committee North West - Cheshire
HRA NRES Centre North West
Barlow House
3rd Floor
4 Minshull Street
Manchester
M1 3DZ

Telephone: 0161 625 7816
Facsimile: 0161 625 7299

18 September 2012

Professor Helen Cooper
Research and Development
Alder Hey Children's NHS Foundation Trust
Eaton Road
Liverpool
L12 2AP

Dear Professor Cooper

Title of project: **Service evaluation of the on-line adolescent diabetes needs assessment tool (ADNAT)**

Thank you for seeking advice from the HRA on whether the above project should be classified as research requiring review by a Research Ethics Committee (REC), or as some other type of activity, such as audit or service evaluation.

Review by a REC is required only for research as specified within the Department of Health's *Governance Arrangements for Research Ethics Committees* (available from the Department of Health's website www.dh.gov.uk) or under legislation, such as the Clinical Trials Regulations, Human Tissue Act and Mental Capacity Act.

You provided the following documents for consideration:

Document	Version	Date
Evaluation study protocol		September 2012
End of study		December 2011
Favourable opinion letter: 08/H1002/32		19 May 2008

These documents have been considered by the Chair.

Our leaflet *"Defining Research"* explains how we differentiate research from other activities and is published on the NRES website under **Applications** *is your project research?* Based on the information you have provided, our advice is that the project is not considered to be research according to this guidance. It would appear to be service evaluation, and therefore it does not require ethical review by a Research Ethics Committee.

It is ultimately the responsibility of the sponsor to decide if a project is research or not. You must therefore contact the sponsor/funder/care organisation where the work will be undertaken to ensure that they agree with this advice.

If you, your sponsor/funder or any care organisation feels that the project should be managed as research and/or that ethical review by a REC is essential, please write setting out your reasons and we will be pleased to consider further.

You should also check with the University of Chester what other review arrangements or sources of advice apply to projects of this type. Guidance may be available from the clinical governance office.

Although ethical review by a REC is not considered necessary in this case, all types of study involving human participants should be conducted in accordance with basic ethical principles such as informed consent and respect for the confidentiality of participants. When processing personal information there are also legal requirements under the Data Protection Act 2000. When undertaking an audit or service/therapy evaluation, the investigator and his/her team are responsible for considering the ethics of their project with advice from within their organisation. University projects may require approval by the university ethics committee.

This letter should not be interpreted as giving a form of ethical approval or any endorsement of the project, but it may be provided to a journal or other body as evidence that ethical approval is not required under the Department of Health's Research Governance Framework for Health and Social Care.

Where care organisations have clarified that a project is not to be managed as research, the Research Governance Framework states that it should not be presented as research within the NHS.

Yours sincerely

Miss Diane Catterall
Committee Co-ordinator

E-mail: diane.catterall@northwest.nhs.uk

Copy to: Dr Mark Helsden, University of Chester (m.helsdon@chester.ac.uk)
